# Vitamin D Status and the Risk for Hospital-Acquired Infections in Critically Ill Adults: A Prospective Cohort Study

**DOI:** 10.1371/journal.pone.0122136

**Published:** 2015-04-07

**Authors:** Jordan A. Kempker, Kathryn G. West, Russell R. Kempker, Oranan Siwamogsatham, Jessica A. Alvarez, Vin Tangpricha, Thomas R. Ziegler, Greg S. Martin

**Affiliations:** 1 Division of Pulmonary, Allergy, Critical Care and Sleep Medicine, Emory University School of Medicine, 615 Michael Street, Suite 205, Atlanta, GA 30322, United States of America; 2 Emory University School of Medicine, Atlanta, United States of America; 3 Division of Infectious Diseases, Emory University School of Medicine, Atlanta, United States of America; 4 Division of Endocrinology, Metabolism and Lipids, Emory University School of Medicine, Atlanta, United States of America; 5 Division of Endocrinology, Metabolism and Lipids, Atlanta VA Medical Center & Emory University School of Medicine, Atlanta, United States of America; D'or Institute of Research and Education, BRAZIL

## Abstract

**Introduction:**

To identify patient characteristics associated with low serum 25-hydroxyvitamin D (25(OH)D) concentrations in the medical intensive care unit (ICU) and examine the relationship between serum 25(OH)D and the risk for hospital-acquired infections.

**Methods:**

This is a prospective observational cohort of adult patients admitted to the medical ICU at an urban safety net teaching hospital in Atlanta, Georgia from November 1, 2011 through October 31, 2012 with an anticipated ICU stay ≥ 1 day. Phlebotomy for serum 25(OH)D measurement was performed on all patients within 5 days of ICU admission. Patients were followed for 30 days or until death or hospital discharge, whichever came first. Hospital-acquired infections were determined using standardized criteria from review of electronic medical record.

**Results:**

Among the 314 patients analyzed, 178 (57%) had a low vitamin D at a serum 25(OH)D concentration < 15 ng/mL. The patient characteristics associated with low vitamin D included admission during winter months (28% vs. 18%, P = 0.04), higher PaO2/FiO2 (275 vs. 226 torr, P = 0.03) and a longer time from ICU admission to study phlebotomy (1.8 vs. 1.5 days, P = 0.02). A total of 36 (11%) patients were adjudicated as having a hospital-acquired infection and in multivariable analysis adjusting for gender, alcohol use, APACHE II score, time to study phlebotomy, ICU length of stay and net fluid balance, serum 25(OH)D levels < 15 ng/mL were not associated with risk for hospital-acquired infections (HR 0.85, 95% CI 0.40-1.80, P = 0.7).

**Conclusions:**

In this prospective, observational cohort of adults admitted to a single-center medical ICU, we did not find a significant association between low 25(OH)D and the risk for hospital-acquired infections.

## Introduction

Vitamin D is a secosteroid hormone which is classically known for its role in bone health and calcium homeostasis [1]. Scientific work within the past decades has uncovered additional extra-skeletal roles of vitamin D in the functioning of the immune system. The vitamin D receptor (VDR) and 1-alpha-hydroxylase, the enzyme that activates vitamin D, have been found in a variety of cells, including cells of the immune system [2]. More specifically, vitamin D is integral to the innate immune system’s production of antimicrobial peptides, such as cathelicidin, in response to pathogens [3]. Cathelicidin is produced by phagocytic leukocytes, mucosal epithelium and keratinocytes, and is present in mucosal secretions and plasma [4]. Furthermore, cathelicidin has been shown to have direct microbicidal effects on various bacterial pathogens, disrupt bacterial biofilms, promote phagocytosis and reactive oxygen species production and induce chemotaxis of other immune cells to sites of infection [4,5]. The cathelicidin-upregulating properties of vitamin D have been demonstrated to have efficacy in *in vitro* studies of human airway and bladder pathogens [6,7].

Clinical research has examined vitamin D’s effects on the immune system primarily in studies on the prevention of respiratory infections, with observational studies showing associations between low vitamin D and the incidence and severity of respiratory infections among community-dwelling adults [8–12]. Among hospitalized patients, there are also associations between low vitamin D and both *Clostridium difficile* and bloodstream infections [13,14]. In critically ill patients, while studies have demonstrated provocative associations between low vitamin D and increased morbidity and mortality, its relationship with sepsis and bloodstream infections upon admission has been inconsistent [15–22]. Examining the risk of hospital-acquired infections, two studies have demonstrated associations with low vitamin D in surgical patients, yet to our knowledge, this question has not been studied in the general medical intensive care unit (ICU) [23,24]. With up to 79% of ICU patients with low vitamin D and a recent randomized controlled trial demonstrating the safety of vitamin D replacement in this population, this could be a potential corrigible risk factor for hospital-acquired infections in a vulnerable population and requires further study [16,18,25,26].

The aims of the current study were to identify the patient characteristics associated with low vitamin D in the medical ICU and to examine the independent relationship between low vitamin D, assessed by serum 25(OH)D concentration, and the risk for hospital-acquired infections. Our hypothesis was that in adults admitted to the medical ICU, an admission serum 25(OH)D concentration < 15 ng/mL would be associated with an increased risk of hospital-acquired infections. While there is no consensus in the medical literature as to the serum 25(OH)D level that is optimal for immune function, this cutoff was chosen for its precedence in the critical care literature and from review of the optimal vitamin D levels for bone health by the 2010 Institute of Medicine Report [15,17,27,28].

## Materials and Methods

A prospective observational cohort design was utilized for this study. Patients admitted to the medical ICU at Grady Memorial Hospital in Atlanta, Georgia from November 1, 2011 through October 31, 2012 were screened for enrollment. Subjects were screened using the ICU census of the electronic medical record and were eligible if they were ≥ 18 years of age and were anticipated to have an ICU stay ≥ 1 day. The anticipated length of stay was determined by study investigators in conjunction with the assessment and plan of the admission documentation. Subjects were enrolled if they or a surrogate gave informed consent or they qualified for a waiver of consent. Subjects were excluded if they were previously enrolled in this study or the study staff was unable to perform phlebotomy within 5 days after ICU admission.

The primary outcome was the development of a hospital-acquired infection, which was defined as an infection during the index hospitalization that was not present within the first 48 hours of admission to the ICU and qualified for an infection of the skin and soft tissue, lower respiratory tract, urinary tract, blood stream or gastrointestinal tract using the 2008 criteria from the Centers for Disease Control and National Healthcare Safety Network (S1 Appendix) [29]. Infections were captured only during the index hospitalization, were determined from the laboratory data and clinical documentation in the electronic medical record, and were adjudicated by an Infectious Diseases specialist on the study team. The secondary outcomes collected were the initiation and duration of mechanical ventilation, hospital and ICU lengths of stay and hospital mortality at 30 days.

The primary exposure of serum 25(OH)D concentration was determined from blood drawn within 5 days of admission to the ICU by trained study staff, preferentially from a central venous catheter or arterial line, or by peripheral phlebotomy if these were unavailable. Assays for serum 25(OH)D were performed using an automated chemiluminescent technique (Automated IDS-iSYS System, Immunodiagnostic Systems, Fountain Hills, AZ) with low vitamin D defined as a concentration < 15 ng/mL. Quality assurance of the serum 25(OH)D determinations was provided by participation in the vitamin D external quality assessment scheme and the NIST/NIH Vitamin D Metabolites Quality Assurance Program. There was no routine practice or protocol for vitamin D status assessment and replacement in our medical ICU so this information was not gathered.

The other information collected from the electronic medical record included patient demographics, ICU admitting diagnoses, past medical and social histories, and physiologic data. Medical comorbidities were captured if they were documented in the past medical history of the hospital record and then categorized into 8 categories. Admitting diagnoses captured if listed in the assessment and plan of the admitting history and physical and then categorized into 5 categories. The details of both categorizations are listed in our case report form available in the Supplementary Information (S1 Appendix). Physiologic data was gathered from the 6 hours before through the 18 hours after the time of the ICU admit orders and selected as the worse value by the Acute Physiology and Chronic Health Evaluation II scoring system [30]. Culture results were recorded as the final result listed in the electronic medical record. Winter season was defined as ICU admission within the months of December through February.

All statistical analyses were performed using SAS 9.3 (SAS Institute: Cary, NC) with a P< 0.05 considered statistically significant. Bivariate analyses were performed using pooled t-tests for continuous variables of normal distribution and equal variances, unpooled t-tests for those with unequal variances and Wilcoxon rank sum tests for those with non-normal distributions. Chi-squared and fisher’s exact tests were used for all categorical variables. The multivariable analysis was performed using a Cox Proportional Hazards model for the time to hospital-acquired infection with subjects censored for death, discharge or at 30 days, whichever came first. The time zero was defined as 2 days after ICU admission since our primary outcome was defined as infections that developed >2 days after ICU admission, marking this as the start of the risk period. The model was built using the purposeful selection of covariates described by Hosmer and Lemeshow and the bivariate analyses by vitamin D status and hospital-acquired infection status are reported in Table 1 [31]. Since we considered death to be a competing risk, we additionally performed a Kaplan-Meier analysis of hospital mortality by 25(OH)D status and a cumulative incidence analysis for hospital-acquired infections that took into account death as a competing risk.

**Table 1:** Patient Characteristics by Vitamin D Status and Hospital-Acquired Infection Statusin Patients Admitted to the Medical Intensive Care Unit at Grady Memorial Hospital, Atlanta, GA November 1, 2011-October 31, 2012.

**Variable**	**25(OH)D ≥ 15 ng/mL N = 136**	**25(OH)D < 15 ng/mL N = 178**	**P**	**No Hospital-Acquired Infection N = 278**	**Hospital-Acquired Infection N = 36**	**P**
**Demographics**						
Age, mean (SD)	57.2 (15.5)	54.4 (14.6)	0.1	55.4 (15.4)	57.3 (11.9)	0.5
Age Categories, n(%)			0.2			0.7
18–44	25 (18)	38 (21)		57 (20.5)	6 (16.7)	
45–54	30 (22)	51 (29)		74 (26.6)	7 (19.4)	
55–64	41 (30)	46 (26)		75 (27.0)	12 (33.3)	
65–74	21 (15)	31 (17)		44 (15.8)	8 (22.2)	
≥ 75	19 (14)	12 (7)		28 (10.1)	3 (8.3)	
Female, n (%)	55 (40)	76 (43)	0.7	115 (41.4)	16 (44.4)	0.7
Weight, kg, mean (SD)	80.8 (22.9)	85.2 (33.2)	0.2	84.1 (28.6)	76.9 (33.2)	0.2
Race/Ethnicity, n (%)			0.8			0.5
Black	110 (81)	151 (85)		234 (84.2)	27 (75.0)	
White	20 (15)	19 (11)		32 (11.5)	7 (19.4)	
Hispanic	5 (4)	7 (4)		10 (3.6)	2 (5.6)	
Asian	1 (1)	1 (1)		2 (0.7)	0	
History of Tobacco Use, n (%)	62 (46)	82 (46)	0.8	127 (45.7)	17 (47.2)	0.2
History of Alcohol Abuse, n(%)	26 (19)	45 (25)	0.4	60 (21.6)	11 (30.6)	0.1
**Past Medical History n(%)**						
Liver disease	7 (5)	16 (9)	0.2	19 (6.8)	4 (11.1)	0.4
Pulmonary disease	37 (27)	36 (20)	0.2	64 (23.0)	9 (25.0)	0.8
Heart disease	98 (72)	129 (72)	0.9	78 (28.1)	9 (25.0)	0.7
Renal Disease	32 (24)	37 (21)	0.6	61 (21.9)	8 (22.2)	1.0
Immunosuppression	25 (18)	26 (15)	0.4	43 (15.5)	8 (22.2)	0.3
Diabetes Mellitus	41 (30)	55 (31)	0.9	87 (31.3)	9 (25.0)	0.4
Hypertension	90 (66)	102 (57)	0.1	172 (61.9)	20 (55.6)	0.4
Cerebrovascular disease	20 (15)	20 (11)	0.4	34 (12.2)	6 (16.7)	0.5
**Admission Data n(%)**						
Winter	24 (18)	49 (28)	0.04	63 (22.7)	10 (27.8)	0.5
Primary Admission Diagnoses, n (%)			0.2			0.1
Respiratory	47 (35)	45 (25)		76 (27.3)	16 (44.4)	
Cardiac	49 (36)	75 (42)		113 (40.7)	12 (33.3)	
Neurological	17 (13)	22 (12)		33 (11.9)	6 (16.7)	
Gastroenterological	12 (9)	12 (7)		23 (8.3)	1 (2.8)	
Other	11 (8)	24 (13)		33 (11.9)	1 (2.8)	
Sepsis at Admission, n (%)	72 (53)	98 (55)	0.7			
**Worst Physiologic parameters within 24 hours of ICU Admission, Mean (SD)**						
PaO_2_/FiO_2_, torr	226(142)	275(243)	0.03	262.2 (165.9)	198.3 (23.0)	0.05
Creatinine, mg/dL	2.3 (2.5)	2.5 (2.7)	0.5	2.5 (2.7)	1.7 (1.2)	<0.01
Mean Arterial Pressure, mm Hg	77 (37)	81 (40)	0.4	80.9 (39.8)	68.3 (26.1)	0.01
Patient on vasopressor therapy, n(%)	35 (26)	48 (27)	0.8	72 (25.9)	11 (30.6)	0.6
Total Bilirubin, mg/dL	1.0 (2.3)	1.6 (3.1)	0.1	1.3 (2.9)	1.1 (1.6)	0.5
Platelet count, x 10^9^/L	193 (105)	189 (159)	0.8	185.3 (96.1)	237.7 (312.6)	0.3
White Blood Cells, x10^9^/L	12.3 (10.4)	11.9 (7.7)	0.7	10.4 (4.7)	12.3 (9.3)	0.06
Hematocrit %	30.8 (8.7)	31.1 (8.1)	0.7	31.0 (8.4)	31.0 (8.1)	1.0
Lactate, mmol/L	3.1 (2.4)	3.4 (2.4)	0.4	3.2 (2.4)	3.8 (2.6)	0.3
Glasgow-Coma Scale score	10 (7–15)	12.5 (7–15)	0.2	12 (7–15)	9 (7–14	0.2
Net fluid balance, L	-0.05 (2.1)	-0.08 (2.4)	0.9	-0.1 (2.3)	-0.1 (1.8)	0.9
SOFA score	7.1 (3.7)	7.1 (3.8)	1.0	6.9 (3.7)	8.5 (3.7)	0.03
APACHE II score	27.5 (7.0)	28.0 (6.9)	0.6	27.8 (7.1)	27.3 (5.9)	0.7
**Days from Hospital Admission to serum 25(OH)D draw**						
Mean (SD)	1.5 (1.1)	1.8 (1.2)	0.02	1.6 (1.1)	2.2 (1.2)	<0.01
**Serum 25-hydroxyvitamin D**						
Mean (SD)	NA	NA		15.5 (9.8)	15.3 (10.4)	0.9
**Categorical Variable, n(%)**						1.0
≥ 30 ng/mL	NA	NA		20 (7.2)	2 (5.6)	
20–29 ng/mL	NA	NA		49 (17.6)	7 (19.4)	
10–19 ng/mL	NA	NA		121 (43.5)	16 (44.4)	
<10 ng/mL	NA	NA		88 (31.7)	11 (30.6)	
**Dichotomous Categorizations, n(%)**						
<20 ng/mL	NA	NA		209 (75.2)	27 (75.0)	1.0
<15 ng/mL	NA	NA		156 (56.2)	22 (61.1)	0.6
<10 ng/mL	NA	NA		88 (31.7)	11 (30.6)	0.9

N = 314.

* 25(OH)D = serum 25α-hydroxyvitamin D; APACHE II = acute physiology and chronic health evaluation 2; SOFA = Sequential Organ Function Assessment

Based on review of prior literature an initial sample size calculation assumed that approximately 30% of the cohort would experience a hospital-acquired infection and that we would observe a 10% absolute reduction in the high 25(OH)D group [32,33]. With an alpha of 0.05 and a power of 0.80, this required a total sample size of 580. However, an interim analysis revealed that our incidence of hospital-acquired infections was significantly lower than anticipated and that we would not be able to reach enough sample size to attain adequate statistical power to show differences at such a low baseline infection rate. Given limited resources we decided to stop the study at one year.

### Ethics Statement

The Institutional Review Board of Emory University and the Grady Research Oversight Committee approved the study (IRB study number 51263). Patients underwent written informed consent if they were able. If patients were unable to provide consent, then a patient surrogate provided informed consent in person or over the telephone. If no surrogate was able to be contacted within 24 hours of our first attempt at contact, we were approved to waive informed consent for these subjects given that the study did not incur procedures that were deemed to be above minimal harm or risk.

## Results

**Fig 1:**
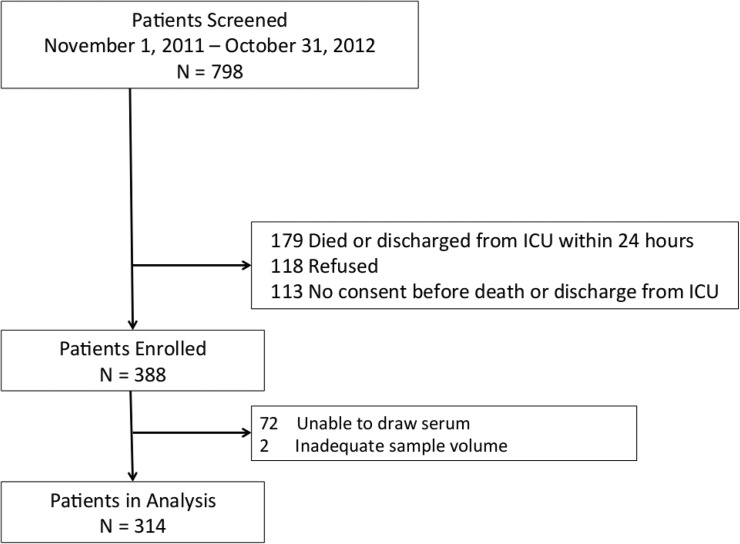
Flowchart of Study Enrollment.

**Fig 2:**
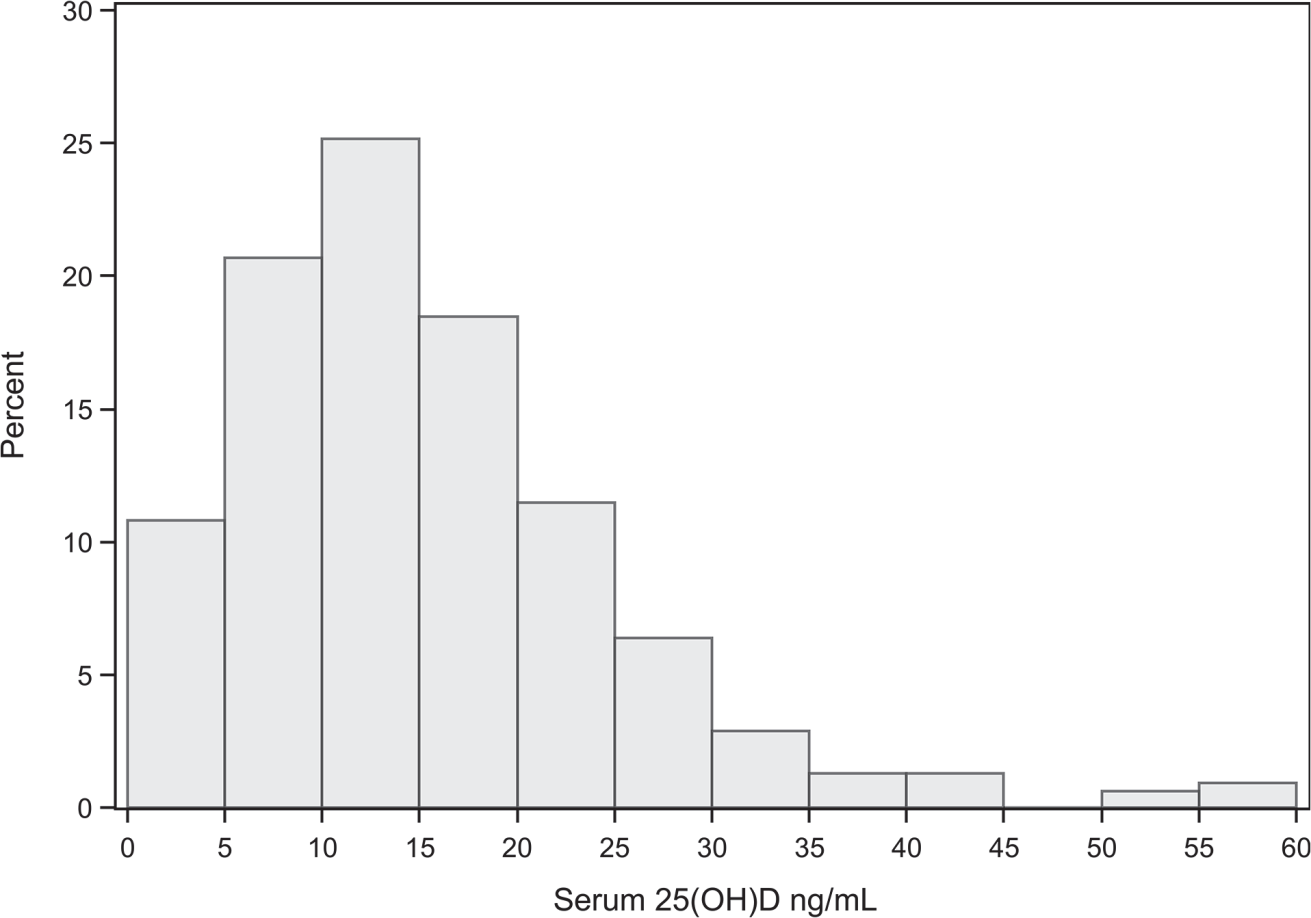
Distribution of Serum 25α-hydroxyvitamin D Concentrations. N = 314.

A total of 798 subjects were screened with 316 enrolled. Two subjects were withdrawn for an inadequate blood collection volume, leaving a final study cohort of 314 subjects (Fig 1). The majority of the study population was male (58%), Black (83%) and between the ages of 45 and 64 years (54%). The most common (40%) primary reason for admission to the ICU was a cardiovascular diagnosis (including circulatory shock) and the majority of patients (58%) were treated for a suspected infection upon admission. Fifty-seven percent of the entire population had a serum 25(OH)D < 15 ng/mL with a mean and median 25(OH)D of 15.4±9.8 ng/mL and 13.4 ng/mL (interquartile range 8.7–19.9 ng/mL), respectively (Fig 2). Mean time from ICU admission to study blood draw was 1.7 days.

### Patient Characteristics by Vitamin D Status

There were no significant differences in the demographic characteristics or underlying medical comorbidities of those with and without low 25(OH)D on admission to the ICU. Patients with low 25(OH)D were more likely to be admitted during the winter season (28% vs. 18%, P = 0.04) and have a longer time from ICU admission to study phlebotomy (1.8 days vs. 1.5 days, P = 0.02). A higher PaO_2_/FiO_2_ (275 torr vs. 226 torr, P = 0.03) was the only physiologic variable on admission associated with low 25(OH)D. Both groups had similar APACHE II and SOFA scores and similar net fluid balances (Table 1).

### Patient Outcomes by Vitamin D Status

A total of 36 adjudicated hospital-acquired infections were documented, with an average time from 48 hours after ICU admission to infection of 7.9±8.1 days. The most common site of infection was the respiratory tract (44%) followed by the genito-urinary tract (25%), blood stream (22%), and gastrointestinal tract (8%). Infections with identified organisms were most often polymicrobial. The most common monomicrobial infecting organism was *Enterococci spp*. followed by *Staphylococcus aureus* (Table 2). There was a significant difference in the distribution of sites of infection by vitamin D status, represented by more bloodstream infections in the low vitamin D group (Table 3).

**Table 2:** Hospital-Acquired Infections by Site of Infection and Organism.

	**N (%)**
**Overall Hospital-Acquired Infections**	36
**Site of Hospital-Acquired Infections**	
Respiratory	16 (44)
Genito-urinary	9 (25)
Blood Stream	8 (22)
Gastroenterological	3 (8)
**Organism** *	
**Unknown**	8 (22)
**Gram-positive**	
Enterococci spp.	5 (14)
Staphylococcus aureus	5 (14)
Clostridium dificile	5 (14)
Coagulase negative staphylococcus	1 (3)
Group B streptococcus	1 (3)
**Gram-negative**	
Pseudomonas aeruginosa	4 (11)
Acinetobacter baumanii	4 (11)
Escherichia coli	3 (8)
Serratia spp.	3 (8)
Enterobacter cloacae	2 (6)
Klebsiella pneumoniae	1 (3)
Citrobacter spp.	1 (3)
**Fungal**	
Candida parapsilosas	1 (3)
Candida glabrata	1 (3)
Candida albicans	1 (3)

* Eight infections were polymicrobial, therefore each percent represents the percent of the total 36 infected patients that had the given organism and will sum to over 100%.

**Table 3:** Patient Outcome by Vitamin D Status in Patients Admitted to the Medical Intensive Care Unit at Grady Memorial Hospital, Atlanta, GA November 1, 2011-October 31, 2012.

**Variable**	**25(OH)D** * **≥ 15 ng/mL N = 136**	**25(OH)D < 15 ng/mL N = 178**	**P**
Mechanically Ventilated, n(%)	76 (56)	100 (56)	1.0
Hospital Length of Stay, median (IQR)	9.5 (4–18)	11 (5–18)	0.3
ICU Length of Stay, median (IQR)	3 (1–8)	4 (2–7)	0.1
Days of Mechanical Ventilation, median (IQR)	4 (1–9)	4 (2–7.5)	1.0
Hospital-Acquired Infection, n(%)	14 (10)	22 (12)	0.6
Site of Infection, n(%)*			0.03
Respiratory	7 (50)	9 (41)	
Genito-urinary	4 (29)	5 (23)	
Blood Stream	2 (14)	6 (27)	
Gastroenterological	1 (7)	2 (9)	
Hospital Mortality, n(%)	18 (13)	31 (17)	0.3

N = 314

* 25(OH)D = serum 25α-hydroxyvitamin D

**Fig 3:**
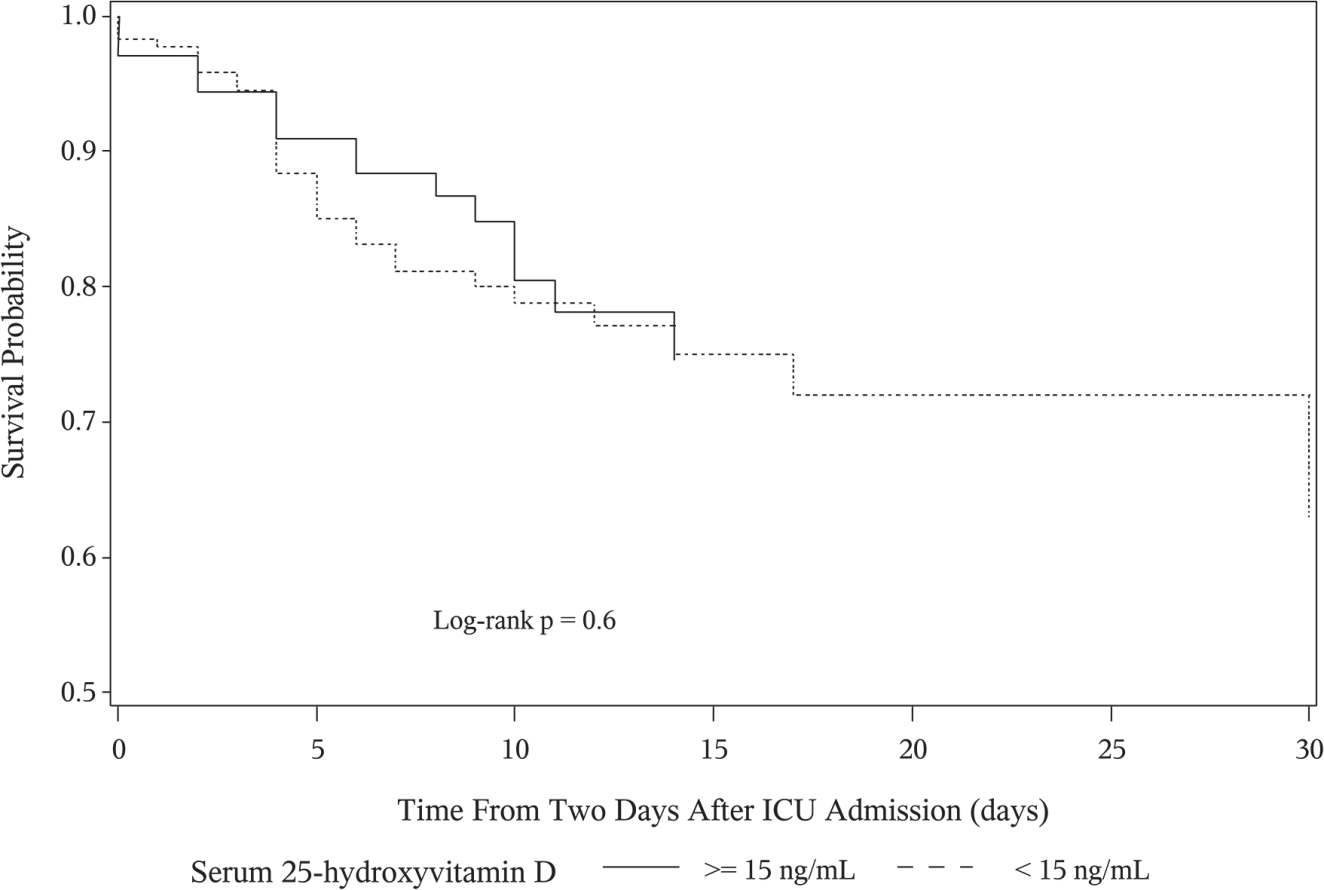
Adjusted Cox Proportional Hazards Curves for Hospital-Acquired Infections by Vitamin D Status. **N = 314.** These curves are adjusted for gender, alcohol use history, APACHE II score, days from ICU admission to study phlebotomy, ICU length of stay and net fluid balance. (APACHE II = acute physiology and chronic health evaluation 2; hospital-acquired infections = Hospital-Acquired Infection; ICU = intensive care unit.)

**Fig 4:**
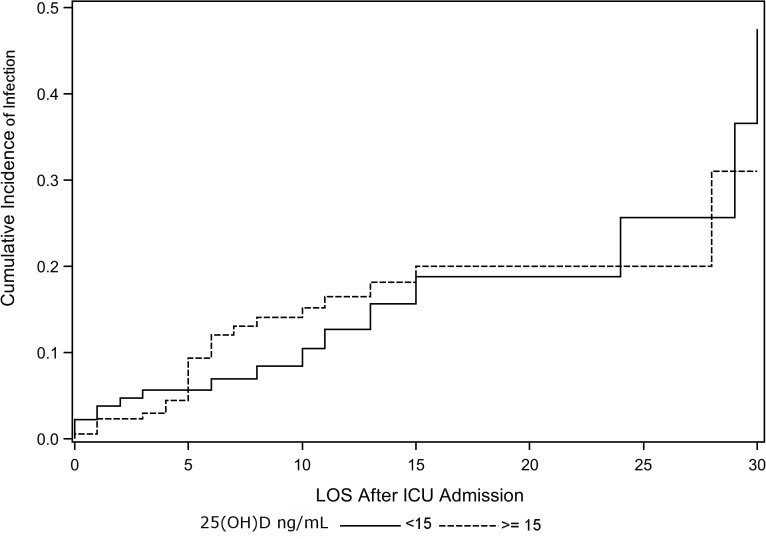
Cumulative Incidence Function For Hospital-Acquired Infection by Vitamin D Status and Accounting For Competing Risk of Hospital Mortality. (ICU = intensive care unit; LOS = length of stay.)

In the unadjusted Cox Proportional Hazards model, serum 25(OH)D < 15 ng/mL was not associated with an increased risk for hospital-acquired infections (HR 1.03, 95% CI 0.53–2.02, P = 0.9). In the multivariable model adjusting for gender, alcohol use history, APACHE II score, days from ICU admission to study phlebotomy, ICU length of stay and net fluid balance, a serum 25(OH)D concentration < 15 ng/mL was not associated with an increased risk for a hospital-acquired infection (HR 0.85, 95% CI 0.40–1.80, P = 0.7) (Fig 3 and S1 Table). The same multivariable analysis was performed with serum 25(OH)D cutoff at <10 ng/mL and <20 ng/mL, revealing no association between 25(OH)D and hospital-acquired infections (HR 0.80, 95% CI 0.39–1.89, P = 0.3 and HR 0.79, 95% CI 0.35–1.80, P = 0.1, respectively). Furthermore, a sensitivity analysis examining the effect of sepsis on ICU admission on the relationship between 25(OH)D and hospital-acquired infection did not reveal any interaction (Tables A, B and C in S2 Table). Finally, a cumulative incidence function accounting for the potentially competing risk of death, did not show an association between low 25(OH)D and hospital-acquired infections (Gray’s Test P = 1.0) (Fig 4).

**Fig 5:**
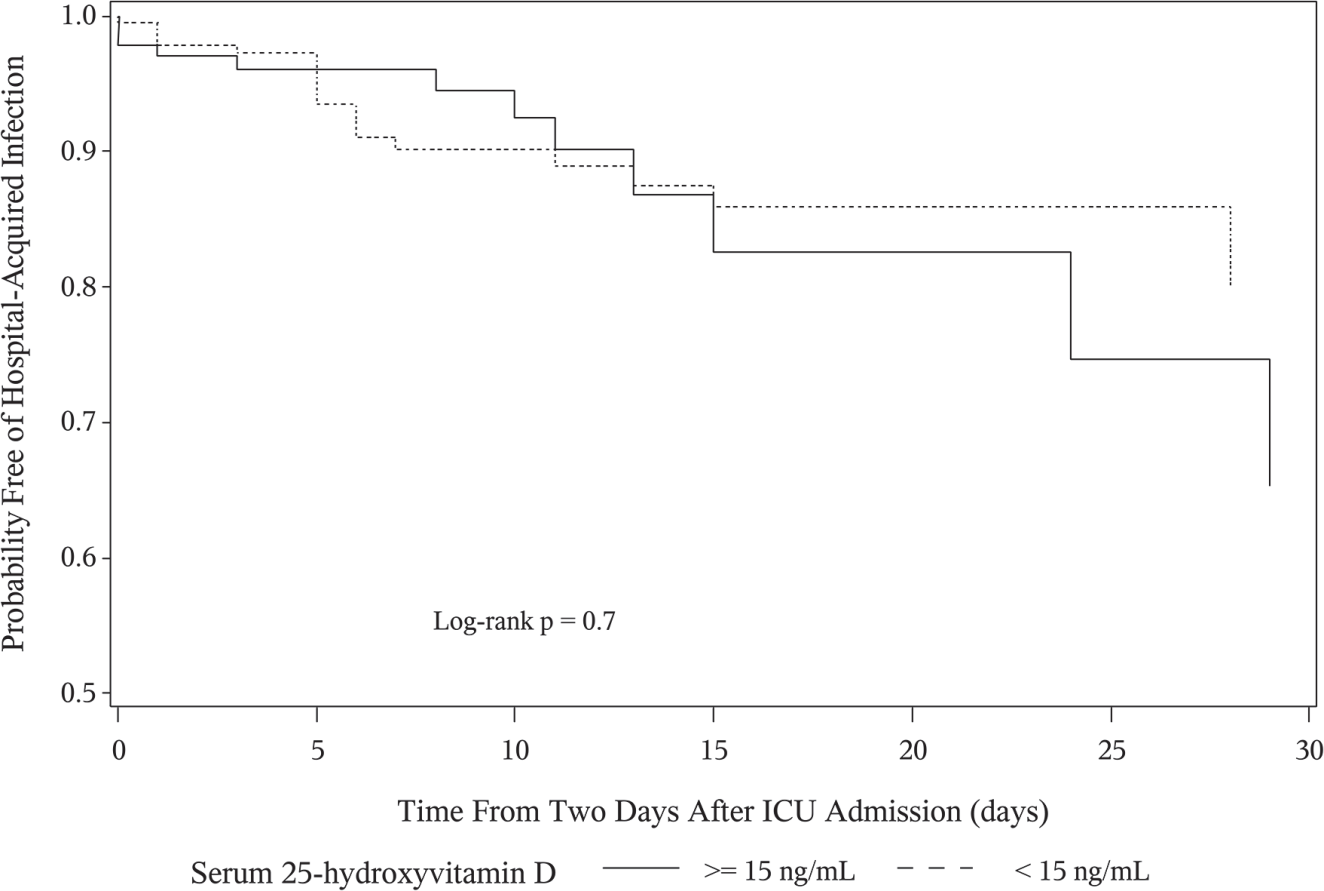
Kaplan-Meier Survival Curves by Vitamin D Status.

In regards to our secondary outcomes, a low 25(OH)D was not associated with hospital mortality, hospital length of stay, ICU length of stay or initiation or duration of mechanical ventilation (Table 3). Kaplan-Meier survival curves did not demonstrate an effect of time on the association between low 25(OH)D and hospital mortality (Fig 5).

## Discussion

To our knowledge, this is the first prospective study to examine the relationship between serum 25(OH)D concentration and the risk for hospital-acquired infections in patients admitted to the medical ICU. Overall, we found a high prevalence of low 25(OH)D, with a 57% of patients having a serum 25(OH)D < 15 ng/mL, and a low rate of hospital-acquired infections, with 11% of all subjects developing a hospital-acquired infection within 30 days. We found no association between low serum 25(OH)D concentrations and the development of a hospital-acquired infection.

The prevalence of low 25(OH)D in our study population was on the higher end of the spectrum of previously published reports when compared other studies that assessed 25(OH)D on admission to the ICU. At a cutoff of a serum 25(OH)D < 20 ng/mL, 3 studies have reported a prevalence of low 25(OH)D between 54–63% and 2 studies between 78–79% [19,21,25,34,35]. In comparison, 75% of our patients had a serum 25(OH)D < 20 ng/mL. While one may speculate whether this was due to the majority of our patients being Black, we did not find an association between race and 25(OH)D status (Table 1). The only characteristic among demographics, past medical histories and admitting diagnoses that we found to be associated with low 25(OH)D was admission during the winter season (Table 1). Among the physiologic measurements, we observed that those with low 25(OH)D had a higher PaO_2_/FiO_2_, indicative of better gas exchange. This may have been due to the trends towards a lower prevalence of pulmonary disease and admission for a respiratory cause in the low 25(OH)D group. However, as these two findings were not statistically significant this explanation remains speculative.

In regards to our primary endpoint, the study findings did not support our *a priori* hypothesis that low 25(OH)D status upon admission to the medical ICU would be a risk factor for hospital-acquired infections. Only a few other studies have looked at the associations between hospital-acquired infections and low vitamin D status. One study of a surgical ICU population showed a trend towards an increased rate of hospital-acquired infection and another study of patients undergoing gastric bypass surgery showed a strong association with the risk of subsequent hospital-acquired infection [23,24]. Additionally, while a low pre-hospital 25(OH)D has been associated in one study with an increased risk for hospital-acquired *Clostridium difficile* infections the results of several other studies have been inconsistent regarding bloodstream infections and sepsis [13–15,19,20,22,36]. Finally, among the secondary outcomes of a recent randomized controlled trial, vitamin D supplementation of critically ill adults showed trends towards a reduction in blood culture positivity among those with an admission serum 25(OH)D concentrations < 12 ng/mL [18]. These inconsistencies may be due to the difficulties in studying vitamin D status in clinical studies. These difficulties include the timing of the serum 25(OH)D measurement to define vitamin D status, small clinical effect sizes, lack of a true placebo, lack of a direct measurement of vitamin D storage and utilization, and genetic variation in vitamin D action [37].

More specific to our study, serum 25(OH)D concentrations might have been the result of the hemodilute or acute inflammatory state of our ICU patients rather than a reflection of the vitamin D nutriture *per se*. Regarding hemodilution, one small, well-designed study has shown that intravenous fluid administration temporarily lowers the serum concentration of 25(OH)D [38]. This is important to our population since early in ICU care, when we measured 25(OH)D, many patients receive large boluses of intravenous fluids. While our study did not find net fluid balance in the first 24 hours to be significantly associated with vitamin D status (Table 2), this number may not be indicative of the true clinical situation as the fluid administration by paramedics and in the emergency department was often not recorded in the medical record. In regards to the effects of inflammation on the vitamin D axis, several studies have suggested that serum 25(OH)D levels decrease during acute inflammation only to recover a few days later, likely in part due to a decrease in vitamin D binding protein during acute inflammation [39–41]. Therefore, our study’s low 25(OH)D levels may not be indicative of a true vitamin D deficiency that would pose as a risk factor for future infection but rather the clinical state of the patient upon ICU admission. While a measurement of serum 25(OH)D prior to the start of critical illness may be more reflective of a subject’s vitamin D status, this was not feasible in our study design.

In addition to the above measurement issues, serum levels of 25(OH)D may not be indicative of the body’s utilization of vitamin D by the innate immune system. There is an emerging scientific literature in the area of tuberculosis and vitamin D status that has identified genetic variations in the vitamin D receptor, demonstrating variation in the induction of genes of the immune system in response to vitamin D [42–44]. While the data are not conclusive, in tuberculosis these VDR polymorphisms have been associated with serum 25(OH)D concentrations and responses to vitamin D therapy [43,44]. In addition, one small study has shown associations between VDR polymorphisms and acute lower respiratory infections in children [45]. As we did not test the VDR polymorphisms in our study cohort, this could account for significant confounding of our results. Along the lines of determining the body’s true vitamin D status and its effects on immune function, we also were not able to measure the other components of the vitamin D axis or the antimicrobial peptides that may be regulated by vitamin D stores. While the measurement of free serum or local intracellular cathelicidin levels, the metabolites of vitamin D, including 1,25α-dihydroxyvitamin D, 24,25-dihydroxyvitman D, or assays to determine bioavailability of vitamin D may have given us a better understanding of an interaction between 25(OH)D and our patients’ immune function and risk for infection, a recent study suggests that serum 25(OH)D correlates very well with many of these other biochemical constituents [46].

Finally, our study was not powered to detect the small reductions in the observed rate of hospital-acquired infections. We had initially anticipated a baseline infection rate of 30% and had decided that an absolute reduction of 10% would be a reasonable, clinically meaningful finding. However, with the observed overall infection rate much lower than anticipated, we realized that it would not be feasible to enroll enough patients to detect small differences in infection rates between the groups. If we were to assume that our observed higher rates of hospital-acquired infections (12% vs. 10%) among those with low 25(OH)D represented a true finding, then the study would have required over 7,000 patients to reach an 80% power at an alpha of < 0.05.

Despite the above issues, this study has several strengths. Its prospective design allowed for the determination of vitamin D status upon admission to the ICU and followed patients forward for a sufficient time afterwards to determine 25(OH)D’s association with the risk of hospital-acquired infections. Furthermore, strict definitions for infection were used and infections were adjudicated by an Infectious Diseases specialist. We also included a diverse subject population reflective of the pathology seen in this urban hospital medical ICU. While this final point may be seen to introduce additional confounding in including such a broad population, we did not find any associations between admitting diagnosis, low 25(OH)D and hospital-acquired infections.

## Conclusion

In this prospective observational cohort of adults admitted to a single-center medical ICU, we did not find an association between low vitamin D status, defined as a serum 25(OH)D concentration <15 ng/mL, and the risk for a hospital-acquired infection within 30 days. While this may be due to the study’s insufficient power to detect slight differences in already low infection rates, it must also be considered that vitamin D’s biological role in immune function may not be clinically appreciable among a critically ill population where other powerful infection control measures are in place. Future randomized-controlled trials may assist in answering whether vitamin D may have a therapeutic role in mitigating the risk of hospital-acquired infections.

## Supporting Information

S1 Appendix Case Report Form for Vitamin D and Hospital-Acquired Infections in Critically Ill Adults. (DOCX)

S1 Table Full Results of Cox Proportional Hazards Model for Hospital-Acquired Infections. N = 314. (DOCX)

S2 Table Sensitivity Analyses of Patients With and Without Sepsis on Admission to the Intensive Care Unit. Table A. Full Results of Cox Proportional Hazards Model for Hospital-Acquired Infections With Admission with Sepsis Added Into the Model. N = 314. Table B. Full Results of Cox Proportional Hazards Model for Hospital-Acquired Infections in Adults Admitted to the Medical Intensive Care Unit Without Sepsis on Admission. N = 144. Table C. Full Results of Cox Proportional Hazards Model for Hospital-Acquired Infections in Adults Admitted to the Medical Intensive Care Unit With Sepsis on Admission. N = 170.
(DOCX)
