# Supplementary material for: Vitamin D Status and the Risk for Hospital-Acquired Infections in Critically Ill Adults: A Prospective Cohort Study
Source: PLoS One. 2015 Apr 7;10(4):e0122136. doi: 10.1371/journal.pone.0122136 (PMC4388655; doi:10.1371/journal.pone.0122136)
Supplement: S1 Appendix — (DOCX) [file pone.0122136.s001.docx]

**Appendix S1. Case Report Form for Vitamin D and Hospital-Acquired Infections in Critically Ill Adults**

CIN ID #: _________ Alt # ____________

Enroll Date: ____/____/__ Consent Date: / / . Blood Drawn: / / .

Initials of person completing data sheet: _____________

**Demographic Information**

**Complete for all patients who Do Not Refuse (consented or enrolled without proxy or consent)**

Patient Initials Age

Hospital Number DOB / / ______

City, state, zip:___________________________ Phone #­­­­­­­­ ­­­­­­­­­­­­­­­­_______________________________

Gender: ☐ female ☐male

Race:

☐ African-American

☐ Caucasian

☐ Hispanic

☐ Asian

☐ Other

☐ Unknown

**Admission Conditions**

**Admitting Diagnosis (Check all that apply, Circle primary diagnosis)**

Cardiovascular

☐ Shock

☐ Acute myocardial Infarction

☐ Unstable Arrythmia

☐ Cardiac Arrest

☐ Hypertensive Crisis

☐ Heart Failure

Renal

☐ Acute Kidney Injury

(Creatinine up 2x from baseline)

☐ New Hemodialysis

Gastroenterological

☐ GI Bleed

☐ Decompensated Cirrhosis

Pulmonary

☐ Respiratory Failure

☐ Asthma Exacerbation

☐ COPD Exacerbation

Neurological

☐ Coma/Altered Mental Status

☐ Intoxication

☐ CVA

☐ Hepatic Encephalopathy

☐ Drug/EtoH Withdrawal

Endocrinological

☐ DKA

☐ Other:_____________________________________

☐ Emergency post-op OR non-operative candidate

☐ Elective post-op

**Admission Septic Status**

**Source may be documented retrospectively and **organism should be being treated****

☐ ☐ **Infected (**Must document a known or suspected source of infection:)

1. **Primary source: date: ___/___/___**

**☐**Blood ☐Respiratory ☐GU ☐GI ☐Pleura ☐CSF ☐Skin/Soft Tissue ☐Other:__________________

1. **Secondary source: date: ___/___/___**

**☐**Blood ☐Respiratory ☐GU ☐GI ☐Pleura ☐CSF ☐Skin/Soft Tissue ☐Other:__________________

**Organism cultured: _______________________________**

**Comorbid Conditions**

Y N

☐ ☐ EtOH History

- ☐ Absent from Chart

☐ ☐ Tobacco Use

- ☐ Absent from Chart
- # pack-years _______

Y N

☐ ☐ Illicit drug use

- ☐ Absent from Chart
- name(s): ______________________
- ☐ ☐ Tox screen sent
  - If yes, “0” or list: _____________________

Y N

☐ ☐ Liver

☐biopsy-proven cirrhosis ☐portal HTN ☐history of UGIB ☐hepatic failure/encephalopathy/coma

☐ ☐ Pulmonary

☐ COPD ☐Asthma ☐Chronic hypoxia/hypercapnia ☐2^o^ polycythemia ☐Severe pulm HTN

☐ ☐ DM

☐ ☐ HTN

☐ ☐ CAD/CHF

☐ NY Heart Association Class 4

☐ ☐ CVA/stroke

☐ ☐ Chronic Renal Insufficiency

☐ On dialysis

☐ ☐ Immunosuppression

☐HIV/AIDS (CD4 = ) ☐ On ART

☐Chemo ☐ XRT ☐Chronic or high-dose steroids ☐Disease suppressing immune function

Other Past Medical History:

**Objective Data:**

**Worse Value from 6 hours prior to 18 hours after admit orders to the ICU**

**(in Patient Summary)**

Date & time of ICU admit orders (ADT Filter on Order Review): ____/___/_ : __

Weight (actual) kg

**Vitals** (In Comprehensive Flow Sheet)

Temp: ___ ____°C

MAP: _____ __mm Hg

HR: _____ ___ bpm

RR: _____ ___ rpm

**Urine Output:**

☐ ≥ 500mL/24 hr

☐ < 500 mL/24 hr

☐ < 200 ml/24hr

**Drugs**(In Current Medications)

☐ ☐ Vasopressors (record highest dose)

☐ Dobutamine

☐ Dopamine (dose: mcg/kg/min)

☐ Norepinephrine (dose: mcg/min)

☐ epinephrine (dose: mcg/min)

☐ ☐ Antibiotics:


**GCS** (In Complex Assessment Flow Sheet)

**☐** Documented in chart

☐ Performed by study investigator

|  | **6** | **5** | **4** | **3** | **2** | **1** |
| --- | --- | --- | --- | --- | --- | --- |
| **Eye Opening** | - | - | Spontaneous | to speech | To pain | None |
| **Verbal (no ventilator)** | - | Oriented | Confused conversation | Inappropriate words | Incomprehensible sounds | No response |
| **Verbal (Ventilated)** | - | Appears oriented | - | Questionably oriented | - | Generally unresponsive |
| **Best Motor** | obeys | localizes | withdraws | flexes | extends | None |

**GCS Score:**

**Labs**

**Chemistry**

Na:

K:

Cl:

HCO_2:_

Creatinine:

Total Bilirubin:

Lactate:

**ABG**

PaO_2_:

PCO_2_:

FiO_2_:

pH:

**Heme:**

HCT:

WBC:

Platelets: ☐ 50% drop from baseline

**Outcomes at 30 days from Admission**

Hospital Admit Date / /

ICU Admit Date / /

Intubation Date / /

Hospital Discharge Date / /

ICU Discharge Date / /

Extubation Date / /

(off any ventilatory support for 48 hours)

Discharge Status at 30 days or end of hospitalization:

☐ home

☐ Still in ICU

☐ transferred to Intermediate Care

☐ Transferred to General Ward

☐ Transferred to Rehab

☐ Transferred to skilled nursing facility

☐ Other healthcare:

☐ Died**Hospital Acquired Infections at 30 days from ICU admission:**

1. **Infection**

Date of culture: / / .

Date of Positivity: / / .

Organism: .

☐ ☐ Fever > 38°C (within 24hr of culture)

☐ ☐ WBCs > 12,000 or <4,000 (within 24hr of culture)

Site:

**☐ Blood Stream**

☐ ☐ Central Line

☐ # (+) cultures:_____/# total cultures:____

**☐ Lower Respiratory Tract**

☐ ☐ Positive Gram Stain

☐ ☐ CXR done

☐ ☐ New or progressive infiltrate or cavity

☐ ☐ Specimen: sputum, tracheal aspirate, miniBAL, BAL

☐ ☐ Signs: purulent sputum, tachypnea, dyspnea, cough, rales, bronchial breath sounds, oxygen desaturation, increase oxygen requirements, increased ventilator requirements.

**☐ Urinary Tract**

☐ ☐ Specimen: clean catch, straight catheter, foley catheter

☐ ☐ Urine WBCs > 10/mm3

☐ ☐ Symptoms: suprapubic tenderness, dysuria, frequency

**☐** **GI Tract**

☐ ☐ Symptoms: Diarrhea, abd pain

☐ ☐ C. Diff toxin positive

**☐ Skin and Soft Tissue**

☐ Site: .

☐ ☐ Signs: Pustules, purulent drainage, abscess, boils, induration, localized redness, swelling.

**☐ Endocarditis:**

☐ ☐ Blood Culture Positive

☐ ☐ Echo with new vegetation

**Antibiotic Treatment for Hospital-Acquired Infection**

1. Initial Antibiotic:

Start Date/Time:

☐ ☐ Initital choice appropriate for Organism Sensitivity

☐ ☐ Initial choice changed according to Organism Sensitivity

☐ ☐ Not changed and appropriate drug according to sensitivity

1. Initial Antibiotic:

Start Date/Time:

☐ ☐ Initital choice appropriate for Organism Sensitivity

☐ ☐ Initial choice changed according to Organism Sensitivity

☐ ☐ Not changed and appropriate drug according to sensitivity

1. Initial Antibiotic:

Start Date/Time:

☐ ☐ Initital choice appropriate for Organism Sensitivity

☐ ☐ Initial choice changed according to Organism Sensitivity

☐ ☐ Not changed and appropriate drug according to sensitivity

1. Initial Antibiotic:

Start Date/Time:

☐ ☐ Initital choice appropriate for Organism Sensitivity

☐ ☐ Initial choice changed according to Organism Sensitivity

☐ ☐ Not changed and appropriate drug according to sensitivity

1. **Infection**

Date of culture: / / .

Date of Positivity: / / .

Organism: .

☐ ☐ Fever > 38°C (within 24hr of culture)

☐ ☐ WBCs > 12,000 or <4,000 (within 24hr of culture)

Site:

**☐ Blood Stream**

☐ ☐ Central Line

☐ # (+) cultures:_____/# total cultures:____

**☐ Lower Respiratory Tract**

☐ ☐ Positive Gram Stain

☐ ☐ CXR done

☐ ☐ New or progressive infiltrate or cavity

☐ ☐ Specimen: sputum, tracheal aspirate, miniBAL, BAL

☐ ☐ Signs: purulent sputum, tachypnea, dyspnea, cough, rales, bronchial breath sounds, oxygen desaturation, increase oxygen requirements, increased ventilator requirements.

**☐ Urinary Tract**

☐ ☐ Specimen: clean catch, straight catheter, foley catheter

☐ ☐ Urine WBCs > 10/mm3

☐ ☐ Symptoms: suprapubic tenderness, dysuria, frequency

**☐ GI Tract**

☐ ☐ Symptoms: Diarrhea, abd pain

☐ ☐ C. Diff toxin positive

**☐ Skin and Soft Tissue**

☐ Site: .

☐ ☐ Signs: Pustules, purulent drainage, abscess, boils, induration, localized redness, swelling.

**☐ Endocarditis:**

☐ ☐ Blood Culture Positive

☐ ☐ Echo with new vegetation

**Antibiotic Treatment for Hospital-Acquired Infection**

1. Initial Antibiotic:

Start Date/Time:

☐ ☐ Initital choice appropriate for Organism Sensitivity

☐ ☐ Initial choice changed according to Organism Sensitivity

☐ ☐ Not changed and appropriate drug according to sensitivity

1. Initial Antibiotic:

Start Date/Time:

☐ ☐ Initital choice appropriate for Organism Sensitivity

☐ ☐ Initial choice changed according to Organism Sensitivity

☐ ☐ Not changed and appropriate drug according to sensitivity

1. Initial Antibiotic:

Start Date/Time:

☐ ☐ Initital choice appropriate for Organism Sensitivity

☐ ☐ Initial choice changed according to Organism Sensitivity

☐ ☐ Not changed and appropriate drug according to sensitivity

1. Initial Antibiotic:

Start Date/Time:

☐ ☐ Initital choice appropriate for Organism Sensitivity

☐ ☐ Initial choice changed according to Organism Sensitivity

☐ ☐ Not changed and appropriate drug according to sensitivity
