# Supplementary material for: Vitamin D Status and the Risk for Hospital-Acquired Infections in Critically Ill Adults: A Prospective Cohort Study
Source: PLoS One. 2015 Apr 7;10(4):e0122136. doi: 10.1371/journal.pone.0122136 (PMC4388655; doi:10.1371/journal.pone.0122136)
Supplement: S1 Table — (DOCX) [file pone.0122136.s002.docx]

**Table S1. Full Results of Cox Proportional Hazards Model for Hospital-Acquired Infections^[[1]](#footnote-1)^ N = 314**

| **Variable** | **Hazard Ratio (95% CI)** | **P** |
| --- | --- | --- |
|  |  |  |
| Serum 25(OH)D < 15 ng/mL | 0.85 (0.4-1.8) | 0.7 |
|  |  |  |
| Days Study Phlebotomy^[[2]](#footnote-2)^ | 1.04 (0.8-1.4) | 0.8 |
|  |  |  |
| ICU Length of Stay^[[3]](#footnote-3)^ | 1.07 (1.0-1.1) | <0.01 |
|  |  |  |
| APACHE II Score^[[4]](#footnote-4)^ | 1.0 (0.9-1.1) | 0.9 |
|  |  |  |
| Net Fluid Balance^[[5]](#footnote-5)^ | 0.84 (0.7-1.0) | 0.07 |
|  |  |  |
| History of Alcohol Abuse | 0.79 (0.5-1.3) | 0.8 |
|  |  |  |
| Male vs Female | 0.77 (0.4-1.7) | 0.5 |
|  |  |  |

1. Subjects were censored for death, discharge or at 30 days from admission to the intensive care unit. [↑](#footnote-ref-1)
2. Hazard ratio (HR) represents each additional day from ICU admission to study phlebotomy. [↑](#footnote-ref-2)
3. HR represents hazard for each additional day in the intensive care unit. [↑](#footnote-ref-3)
4. HR represents hazard for each additional point in APACHE II score. [↑](#footnote-ref-4)
5. HR represents hazard for each additional Liter in total body fluid balance in the first 24 hours of ICU admission. [↑](#footnote-ref-5)
