# Supplementary material for: Vitamin D Status and the Risk for Hospital-Acquired Infections in Critically Ill Adults: A Prospective Cohort Study
Source: PLoS One. 2015 Apr 7;10(4):e0122136. doi: 10.1371/journal.pone.0122136 (PMC4388655; doi:10.1371/journal.pone.0122136)
Supplement: S2 Table — Table A. Full Results of Cox Proportional Hazards Model for Hospital-Acquired Infections With Admission with Sepsis Added Into the Model. N = 314. Table B. Full Results of Cox Proportional Hazards Model for Hospital-Acquired Infections in Adults Admitted to the Medical Intensive Care Unit Without Sepsis on Admission. N = 144. Table C. Full Results of Cox Proportional Hazards Model for Hospital-Acquired Infections in Adults Admitted to the Medical Intensive Care Unit With Sepsis on Admission. N = 170. (DOCX) [file pone.0122136.s003.docx]

**Sensitivity Analyses of Patients With and Without Sepsis on Admission to the Intensive Care Unit**

**Table S2A. Full Results of Cox Proportional Hazards Model for Hospital-Acquired Infections With Admission with Sepsis Added Into the Model.^[[1]](#footnote-1)^ N = 314**

| **Variable** | **Hazard Ratio (95% CI)** | **P** |
| --- | --- | --- |
|  |  |  |
| Serum 25(OH)D < 15 ng/mL | 0.87 (0.4-1.8) | 0.7 |
|  |  |  |
| Sepsis on Admission | 0.76 (0.3-1.8) | 0.5 |
|  |  |  |
| Days Study Phlebotomy^[[2]](#footnote-2)^ | 1.04 (0.8-1.4) | 0.8 |
|  |  |  |
| ICU Length of Stay^[[3]](#footnote-3)^ | 1.07 (1.0-1.1) | <0.01 |
|  |  |  |
| APACHE II Score^[[4]](#footnote-4)^ | 1.0 (0.9-1.1) | 1.0 |
|  |  |  |
| Net Fluid Balance^[[5]](#footnote-5)^ | 0.84 (0.7-1.0) | 0.08 |
|  |  |  |
| History of Alcohol Abuse | 0.80 (0.5-1.4) | 0.4 |
|  |  |  |
| Male vs Female | 0.78 (0.4-1.7) | 0.5 |
|  |  |  |

**Table S2B. Full Results of Cox Proportional Hazards Model for Hospital-Acquired Infections in Adults Admitted to the Medical Intensive Care Unit Without Sepsis on Admission. N = 144**

| **Variable** | **Hazard Ratio (95% CI)** | **P** |
| --- | --- | --- |
|  |  |  |
| Serum 25(OH)D < 15 ng/mL | 0.94 (0.2-4.1) | 0.9 |
|  |  |  |
| Days Study Phlebotomy | 0.94 (0.5-1.7) | 0.8 |
|  |  |  |
| ICU Length of Stay | 1.20 (1.1-1.3) | <0.01 |
|  |  |  |
| APACHE II Score | 1.03 (0.9-1.2) | 0.7 |
|  |  |  |
| Net Fluid Balance | 0.72 (0.4-1.2) | 0.2 |
|  |  |  |
| History of Alcohol Abuse | 0.39 (0.1-1.4) | 0.2 |
|  |  |  |
| Male vs Female | 0.70 (0.1-3.7) | 0.7 |
|  |  |  |

**Table S2C. Full Results of Cox Proportional Hazards Model for Hospital-Acquired Infections in Adults Admitted to the Medical Intensive Care Unit With Sepsis on Admission. N = 170**

| **Variable** | **Hazard Ratio (95% CI)** | **P** |
| --- | --- | --- |
|  |  |  |
| Serum 25(OH)D < 15 ng/mL | 0.83 (0.3-2.1) | 0.7 |
|  |  |  |
| Days Study Phlebotomy | 0.98 (0.7-1.4) | 0.9 |
|  |  |  |
| ICU Length of Stay | 1.04 (1.0-1.1) | 0.1 |
|  |  |  |
| APACHE II Score | 1.0 (0.9-1.1) | 0.9 |
|  |  |  |
| Net Fluid Balance | 0.86 (0.7-1.1) | 0.2 |
|  |  |  |
| History of Alcohol Abuse | 1.07 (0.5-2.1) | 0.8 |
|  |  |  |
| Male vs Female | 0.66 (0.3-1.6) | 0.4 |
|  |  |  |

1. Subjects were censored for death, discharge or at 30 days from admission to the intensive care unit. [↑](#footnote-ref-1)
2. Hazard ratio (HR) represents each additional day from ICU admission to study phlebotomy. [↑](#footnote-ref-2)
3. HR represents hazard for each additional day in the intensive care unit. [↑](#footnote-ref-3)
4. HR represents hazard for each additional point in APACHE II score. [↑](#footnote-ref-4)
5. HR represents hazard for each additional Liter in total body fluid balance in the first 24 hours of ICU admission. [↑](#footnote-ref-5)
